# Chemokines and lymphocyte homing in Sjögren’s syndrome

**DOI:** 10.3389/fimmu.2024.1345381

**Published:** 2024-04-26

**Authors:** Jiahe Liao, Xinbo Yu, Ziwei Huang, Qian He, Jianying Yang, Yan Zhang, Jiaqi Chen, Weijiang Song, Jing Luo, Qingwen Tao

**Affiliations:** ^1^ Graduate School, Beijing University of Chinese Medicine, Beijing, China; ^2^ Traditional Chinese Medicine Department of Rheumatism, China-Japan Friendship Hospital, Beijing, China; ^3^ Traditional Chinese Medicine Department, Peking University Third Hospital, Beijing, China; ^4^ Beijing Key Laboratory of Immune Inflammatory Disease, China-Japan Friendship Hospital, Beijing, China

**Keywords:** Sjögren’s syndrome, lymphocyte homing, chemokines, addressins, targeting therapy

## Abstract

Sjögren’s syndrome (SS) is a chronic systemic autoimmune disease that typically presents with lymphocyte, dendritic cell, and macrophage infiltration of exocrine gland ducts and the formation of ectopic germinal centers. The interactions of lymphocyte homing receptors and addressins and chemokines and their receptors, such as α4β7/MAdCAM-1, LFA-1/ICAM-1, CXCL13/CXCR5, CCL25/CCR9, CX3CL1/CX3CR1, play important roles in the migration of inflammatory cells to the focal glands and the promotion of ectopic germinal center formation in SS. A variety of molecules have been shown to be involved in lymphocyte homing, including tumor necrosis factor-α, interferon (IFN)-α, IFN-β, and B cell activating factor. This process mainly involves the Janus kinase-signal transducer and activator of transcription signaling pathway, lymphotoxin-β receptor pathway, and nuclear factor-κB signaling pathway. These findings have led to the development of antibodies to cell adhesion molecules, antagonists of chemokines and their receptors, compounds interfering with chemokine receptor signaling, and gene therapies targeting chemokines and their receptors, providing new targets for the treatment of SS in humans. The aim of this study was to explore the relationship between lymphocyte homing and the pathogenesis of SS, and to provide a review of recent studies addressing lymphocyte homing in targeted therapy for SS.

## 1 Introduction

Sjögren syndrome (SS) is a common chronic autoimmune disease, associated with other systemic autoimmune diseases such as systemic lupus erythematosus (SLE) and rheumatoid arthritis (RA), and may be comorbid with them (1). SS mainly affects the exocrine glands, especially salivary glands and lacrimal glands, and primarily presents with dry mouth and eyes (2). In severe cases, patients may show damage to extra-glandular organs and systems, including the dermatologic, musculoskeletal, pulmonary, gastrointestinal, hepatobiliary, hematologic, renal, or neurologic systems (3). The more serious aspect of SS is the considerably higher risk of developing malignant non-Hodgkin’s lymphoma (NHL). It occurs in about 2%-5% cases, and it is the main cause of a decreased survival in SS (4). Pathological manifestations of SS exocrine gland tissue include infiltration of CD4+ T and B lymphocytes, altered follicular structure and fibrosis, hypoplasia or loss of glandular function (5, 6). In addition, approximately 25 ± 5% of SS salivary gland will have the formation of ectopic germinal centers (GCs), a specialized site for B cell activation and antibody maturation in non-lymphoid organs (7, 8).

Chemokines and their receptors play regulatory roles in the inflammatory response, and abnormal expression of chemokines has been shown to be associated with the pathogenesis of various autoimmune diseases, including RA, SLE, and Behcet disease. However, the association between chemokines and the pathogenesis of SS has received less attention (9, 10). Chemokines and their receptors have been recently shown to be involved in the lymphocyte homing process in SS, and are closely related to the generation of ectopic lymphoid structures (ELSs), a specialized microenvironment that forms in response to persistent antigenic and inflammatory stimuli in non-lymphoid organs (11, 12). Expression of chemokines promotes the recruitment of lymphoid tissue inducer cells, causing crosstalk between cells and inducing GCs (13). GCs have been shown to be predictive of an increased risk of lymphoma in SS patients. Investigation of the role of chemokines might give clues for a better understanding of the pathophysiology of mechanisms of SS and of SS-associated lymphomagenesis, leading to the identification of new treatment targets in SS. Therefore, a literature review was performed on SS and homing mechanisms; a total of 168 papers were selected for discussion. The aim of this review is to provide an update concerning the targets of lymphocyte homing in SS.

## 2 Lymphocyte migration molecules

Lymphocyte migration is a form of lymphocyte homing that primarily involves the migration of lymphocytes from the bloodstream to various tissues and organs and is mediated by a series of molecules (14). The chemokines secreted by the cells of various organs in the organism bind to the chemokine receptors expressed by the lymphocytes and promote the binding of the homing receptors on the surface of the lymphocytes to specific addressins, which in turn facilitates the migration of the lymphocytes to the specific tissues and organs. The homing process involves a cascade of migratory molecules, including lymphocyte homing receptors such as selectins/integrins, addressins, chemokines, and their receptors (15). Lymphocyte homing receptors are a class of molecules expressed on the surface of lymphocytes, and include L-selectin, P-selectin, and E-selectin of the selectin family, α4β7 of the integrin family, very late activation antigen VLA-4 (α4β1), and the lymphocyte function-associated antigen LFA-1 (αLβ2), whose interactions with the corresponding addressins mediate the processes of adhesion and efflux between lymphocytes and endothelial cells (16, 17). Addressins are adhesion molecules expressed by vascular endothelial cells, including the immunoglobulin superfamily of mucosal addressin cell adhesion molecule-1 (MadCAM-1), intercellular cell adhesion molecule-1 (ICAM-1), and vascular cell adhesion molecule (VCAM-1), E calreticulin of the calreticulin family, and a number of unclassified adhesion molecules such as mucosal vascular addressin (Mad) and peripheral node addressin (PNAD) (18, 19).

Chemokines are a series of small molecular mass proteins that play important roles in the generation of lymphoid organs under physiological and pathological conditions, as well as in the differentiation, homing, and recirculation of lymphocyte subpopulations in the immune response (20). Many chemokines are associated with lymphocyte homing, and they vary from one tissue site to another. Chemokines are classified into four subclasses, C, CC, CXC, and CX3C, according to the order of the two conserved cysteines at their amino-terminal end, of which CC and CXC are considered to be the two major chemokine subclasses (21). Chemokines can be expressed constitutively and inducibly. The vast majority of constitutive chemokines have specific receptors, whereas inducible chemokines show shared receptors. Each subpopulation of immune cells shows a different pattern of chemokine receptor expression, which allows them to respond differently to chemokines and migrate according to the specific needs of each environment (22). Chemokines and their receptors constitute a complex network widely involved in cellular immunity, growth and development, and inflammation, playing diverse roles in chronic infectious diseases, autoimmune diseases, and tumors (23).

## 3 Bioassays for the determination of chemokines

In recent years, saliva and tears have emerged as promising fluids for the important diagnosis of SS through the detection of chemokine biomarkers therein, which has had a tremendous impact on the development of multiple assay platforms (24). However, bioassays specifically designed to selectively determine one or more chemokines are scarce. Methods to determine chemokine levels are mostly based on immunoassays such as ELISA (25). Western blotting assays have also been used to study the expression of various chemokines in biological fluids (26). However, these techniques have limited sensitivity in detecting very small amounts of target compounds. Most of the immunoassays require long assay times, do not allow real-time study of chemokine secretion, and are also dependent on specific and sensitive immunoreagents, thus not allowing high-throughput analysis of protein expression in tissues (27). Proteomics is a promising tool, and tear proteomics has made relatively less progress than saliva proteomics for technical reasons (28). Salivary proteomics not only identifies chemokine biomarkers, but also helps to elucidate the molecular pathways behind disease pathogenesis, which allows for better stratification of patients and potentially opens up new avenues for targeted therapies (29). However, limitations regarding the necessary technologies for saliva collection and storage and different protocols for proteomic and molecular analysis need to be urgently addressed (30). In addition, electrochemical and optical biosensors have been applied for chemokine determination. Although they are highly sensitive and selective, they are more susceptible to physical damage and environmental interferences, and are more expensive to perform and analyze (31).

## 4 Lymphocytic infiltration of exocrine glands in SS

The histologic features of exocrine glands in SS include the presence of lymphocytic infiltration around ductal cells, with a minority of other immune cells such as macrophages, dendritic cells (DCs), and natural killer (NK) cells (32). Inflammatory lesions usually show focal dense aggregates, and the composition of the lesion varies with the severity of the infiltrate, with T cells predominating in mild infiltrates and B cells in severe infiltrates (33). CD4+ T cells are the main infiltrating T cells, with helper T cell (Th)1, Th2, Th17, and follicular helper T (Tfh) cells (34). Th1 cytokines are represented by interferon (IFN) and tumor necrosis factor (TNF), whose expression has been associated with glandular tissue damage; Th2 cells play a key role in maintaining B cell function; Th17 cells mainly secrete interleukin (IL)-17, which can induce the expression of a variety of cytokines, such as pro-inflammatory cytokines, chemokines, and matrix metalloproteins (MMPs); and Tfh cells are essential for GC formation and B cell activation (35–37). Although CD4+ T cells are a key factor in the immunopathogenesis of SS, various studies have shown that CD8+ T lymphocytes contribute to vesicular damage in exocrine glands (38). CD27+ memory B cells, marginal zone B cells, and plasma cells are all key B cell subpopulations involved in the pathogenesis of SS, and play a role in promoting SS pathogenesis in terms of antigen presentation and cytokines (39).

In summary, the pathological changes in SS are lymphocytic infiltration of exocrine glands, whereas chemokines and their receptors are involved in causing aggregation of lymphoid cells and induction of the chronic inflammatory response. The levels of the chemokines CCL19, CXCL8, CXCL9, CXCL10, CXCL11, and CXCL17 have been shown to be significantly increased in the saliva or tears of SS patients, and those of the chemokines CCL5, CCL17, CCL21, CCL22, CCL25, CXCL1, and CXCL12 have been shown to be upregulated in the salivary glands of SS patients. In addition, both CXCL13 and CX3CL1 levels were elevated in the serum of SS patients. Thus, aberrantly expressed chemokines in SS patients selectively induce and regulate lymphoid cell subsets, which regulate the circulation of lymphocytes between target tissues, blood, and secondary lymphoid organs to form ectopic GCs and participate in their disease processes.

## 5 Lymphocyte homing and SS

Multiple chronic inflammatory diseases and autoimmune diseases such as SS, RA, multiple sclerosis, and Crohn’s disease involve the entry of lymphocytes in the blood into the lymphoid tissues or inflammatory sites. Selectins mediate initial and rolling adhesion is the first step in the process: the mobile lymphocytes initially tether to the vascular wall, and then roll along the wall surface of the blood vessel. After activation by chemokines, the stimulation of cell surface integrins is the second step. Integrins mediate stable adhesion and migration is the third step: activated α4β7, VLA-4, and LFA-1 on the surface of lymphocytes bind tightly to the adhesion molecules MAdCAM-1, VCAM-1, and ICAM-1, respectively, expressed on endothelial cells, which reduces the rolling speed of lymphocytes and promotes the occurrence of firm adhesion, eventually resulting in migration to specific tissues.

### 5.1 Abnormal expression of lymphocyte homing receptor and addressin

#### 5.1.1 L-selectin/PNAD

L-selectin/PNAD L-selectin is a cell adhesion molecule expressed on the surface of naïve and central memory T cells, and is involved in the initial attachment of lymphocytes to high endothelial venules (HEVs) in lymph nodes. PNAD, a sulfated and fucosylated glycoprotein, is expressed on HEVs, and facilitates the entry of naïve T cells into lymphoid organs at non-mucosal tissue sites. L-selectin/PNAD is involved in the tethering and rolling of lymphocytes along the HEV in lymphoid tissues. The salivary levels of soluble L-selectin and IL-7 have been shown to be higher in Indian patients with SS (40). SS patients with higher serum concentrations of circulating L-selectin are more likely to have Raynaud’s phenomenon, autoimmune thyroiditis, and elevated levels of rheumatoid factor (RF) (41).

#### 5.1.2 α4β7/MAdCAM-1

α4β7 belongs to the integrin family, and is expressed on initial T and B lymphocytes, NK cells, monocytes, macrophages, and eosinophils. MAdCAM-1, the predominant ligand for α4β7, is expressed by mucosal endothelial cells of mesenteric lymph nodes, Peyer’s patches, and associated lymphoid tissues (42). α4β7/MAdCAM-1 mediates rolling adhesion of lymphocytes to the capillary lumen on mucosal tissues, followed by stable adhesion to endothelial cells. One study revealed a significant increase in the α4β7-negative cell population in IL-17-expressing cells in the salivary glands, peripheral blood, and spleen of aged NOD mice, and the marked increase in α4β7-negative IL-17-expressing cells in the salivary glands may be involved in the onset and development of SS (43).

#### 5.1.3 VLA-4/VCAM-1

VLA-4 is a heterodimer of α4 and β1, also known as leukocyte integrins or CD49d/CD29, which is expressed on the surface of most lymphocytes, monocytes, eosinophils, and basophils, while VCAM-1 is a member of the immunoglobulin superfamily that is expressed widely in macrophages, DCs, myeloid fibroblasts, and myocytes. VLA-4 selectively promotes stable adhesion of lymphocytes through the specific binding of VCAM-1. Animal experiments verified that the vascular endothelium in the inflamed region of the lacrimal gland in nonobese diabetic (NOD) mice express VCAM-1 and PAND, and that the majority of lymphocytes in the inflamed glands express a4 integrin, L-selectin, and LFA-1 (44). VCAM-1 staining indicated that VCAM-1 may be more important in determining the distribution of B than T lymphocytes in lymphocytic infiltration of non-lymphoid tissue in SS patients, and thus GCs may form by immigration of B cells via VCAM-1+ vessels at the center of T cell aggregates (45).

#### 5.1.4 LFA-1/ICAM-1

LFA-1 is composed of α-chain CD11a and β-chain CD18, which are distributed on the surface of T and B lymphocytes, monocytes, macrophages, and neutrophils, playing important roles in lymphocyte passage through the vascular endothelium. ICAM-1 also belongs to the immunoglobulin superfamily, and is distributed on the surface of fibroblasts, vascular endothelial cells, and activated lymphocytes. LFA-1/ICAM-1 mainly mediates the migration of lymphocytes toward the transvascular endothelium, and their eventual passage through the vascular endothelium to the site of injury. It has shown that in the lacrimal gland of NOD mice, LFA-1 and ICAM-1 are upregulated in lacrimal acinar cells and infiltrating lymphocytes (46). Previous study has also reported that ICAM-1 expression on epithelial cells is moderately upregulated in the salivary gland microenvironment of SS patients (47). Tissue biopsies of salivary glands from SS patients have suggested that cytokine-mediated upregulation of VCAM-1 and ICAM-1 promotes the recruitment of T cells expressing VLA-4 and LFA-1 (48). Further in vitro experiments revealed that TNF-induced apoptotic human salivary gland cells significantly upregulated ICAM-1 and CCL20 expression, and ultimately triggered apoptosis and tissue destruction (49).

### 5.2 Abnormal expression of chemokines and their receptors

#### 5.2.1 CXCL13/CXCR5

CXCL13, a member of the CXC chemokine family, also known as B cell-attracting chemokine-1 (BCA-1) or B lymphocyte chemoattractant (BLC), is the major active chemokine on mature B cells. CXCL13 is constitutively expressed in secondary lymphoid organs such as the spleen and lymph nodes. CXCR5 is the corresponding receptor for CXCL13, and is predominantly expressed on the surface of B and Tfh cells. CXCL13 and CXCR5 are associated with B lymphocytes, Tfh cell homing, and the formation of B cell follicular zones in lymphoid organs. CXCL13 is recognized as a biomarker of SS severity and correlates with disease activity, including clinical parameters. Its expression in human serum and saliva has been proven to increase with disease progression (50), with a marked increase in the number of CXCR5-expressing cells infiltrating focal salivary gland and interstitium (51). CXCL13 has also been identified as a biomarker for histological involvement in SS. Recent studies have shown that elevated serum levels rather than saliva levels of CXCL13 in SS patients are correlated with the histomorphometric features of the salivary glands, including the size of aggregates and the formation of GCs (52, 53), as well as with the risk and incidence of NHL (54, 55). CXCL13 plays a crucial role in lymphocyte neogenesis, maintenance of secondary lymphoid tissue structure, and immune response (56). It is also involved in the initiation and organization of ELSs. In inflammatory tissues, heightened CXCL13 expression regulates the infiltration and localization of B cells, as well as their movement within ectopic GCs (57). Furthermore, CXCL13 can attract Tfh cells towards B cells in ELSs, facilitating B-cell help (58). Collectively, CXCL13 is currently the most extensively studied and most promising chemokine in SS, and may serve as a reliable biomarker for monitoring and diagnosis of SS.

#### 5.2.2 CXCL12/CXCR4

CXCL12 was originally discovered as a pre-B cell growth factor, and is indispensable in lymphangiogenesis. It can be secreted by bone marrow stromal cells, and is therefore also named stromal cell-derived factor-1 (SDF-1). Human CXCL12 is chemotactic for CD4+ T lymphocytes, monocytes, neutrophils and DCs, all of which also express CXCR4. CXCL12/CXCR4 is an important chemokine for the aggregation of B cells into lymphoid follicles, and for the survival of malignant B cells in the salivary glands of mucosa-associated lymphoid tissue (MALT) lymphoma (56). CXCL12 was predominantly observed in ducts and malignant B cells infiltrating the salivary glands of SS patients with MALT lymphoma; levels of CXCL12 were elevated in MALT lymphoma and isolated tumor cells (56). Upregulation of CXCL12, along with elevated CXCR4 expression on CD4+ effector memory T (TEM) cells, has been observed in epithelial cells adjacent to lymphocyte-infiltrated areas of salivary and lacrimal glands in alymphoplasia (aly)/aly mice (59). An *in vitro* Transwell migration assay verified that the migration of (aly)/aly mice CD4+ TEM cells was significantly elevated in response to CXCL12, and the migration response increased with increasing CXCL12 concentration (60).

#### 5.2.3 CCL21/CCR7

CCL21, also known as exodus-2 and secondary lymphoid chemokine (SLC), is a small homeostatic cytokine belonging to the CC chemokine family. Human CCL21 is highly expressed in secondary lymphoid tissues such as the lymph nodes, appendix, and spleen. CCR7 is expressed predominantly on the surface of T cells, B cells, activated NK cells, and DCs, where is it frequently involved in promoting migration. CCL21 binding to a specific receptor, CCR7, synergistically regulates the initiation of immune response and induction of immune tolerance. High levels of CCL21/CCR7 expression in the salivary glands of SS patients were associated with elevated erythrocyte sedimentation rate (ESR), IgG and RF levels, anti-Ro/SSA and La/SSB antibody titers, and a higher focus score and European League Against Rheumatism SS disease activity index (ESSDAI) value on biopsy (61, 62). In patients with extra-glandular manifestations of SS, the prevalence of lymphadenopathy increased with elevated CCL21 levels (63). It has been shown that a strong up-regulation of CCL21 takes place in correlation with the presence of a high number of activated T cells infiltrating the glands, which suggested a prominent role for CCL21 in the SS-associated lymphomagenesis by recruiting and chronically activating T cells, which, once stimulated, provide a stimulus for the expansion and survival of B cells within the reaction zone (56). Hence, CCL21 is directly involved in the construction of ectopic reactive lymphoid tissue.

#### 5.2.4 CCL25/CCR9

CCL25, the thymus-expressed chemokine (TECK), is expressed primarily in the thymus and intestinal epithelial cells, but can also be produced by parenchymal cells, and migrates immature T cells to the thymus for maturation and release. CCL25 is found in the oral mucosa of patients with SS, and is primarily produced by epithelial cells (64). CCL25 binds to the chemokine receptor CCR9, which is expressed on the cell membranes of lymphocytes, monocytes, DCs, and neutrophils. CCR9-expressing cells were observed in the vicinity of epithelial cells in mucosal tissues (65). A study investigating the local and systemic CCL25/CCR9 axis in SS patients found that the levels of CCR9-expressing Th cells and their ligand CCL25 were increased in the salivary glands of SS patients; enhanced CCL25 expression attracted circulating CCR9-expressing Th cells (66). These CCR9-expressing Th cells were highly responsive to IL-7; secreted high levels of IFN-γ, IL-21, and IL-17; and effectively stimulated B cells, confirming that the CCL25/CCR9 axis plays an important role in the immunopathology of SS (65).

#### 5.2.5 CX3CL1/CX3CR1

CX3CL1 is the only member of the CX3C subfamily of chemokines, and its specific endogenous receptor is CX3CR1. CX3CL1 exists in a membrane-bound and a shed form, and the membrane-bound form can be expressed on epithelial cells and vascular endothelial cells (67). It was shown that CX3CL1 induction, cleavage and recruitment of CX3CR1-expressing immune cells were associated with cathepsin S activity, which was significantly and specifically increased in SS patient tears (68). Soluble CX3CL1, which is cleaved from membrane-bound CX3CL1 by proteases including cathepsin S, mediates chemotaxis of CX3CR1 expressing cells. CX3CR1 is predominantly expressed on monocytes, macrophages, neutrophils, T cells, and activated NK cells. CX3CL1/CX3CR1 is closely associated with leukocyte capture and adhesion and chemotaxis of inactivated peripheral CD8+ T cells, NK cells, and monocytes. CX3CL1 levels were found to be significantly elevated in lacrimal gland acinar cells, and the levels of CX3CR1-expressing T cells and macrophages were significantly increased in the lacrimal glands of NOD mice (68). In addition, CX3CL1 protein levels were elevated in the serum of SS patients; transcriptional profiling of peripheral B cells showed upregulation of CX3CR1 expression (69); and histological assessments showed upregulation of the mRNA levels of CX3CL1 and CX3CR1 in the salivary glands of SS patients (70). Moreover, serum CX3CL1 levels were significantly higher in SS patients with extra-glandular manifestations than in those without extra-glandular manifestations (71). As a contributor to the formation of ectopic GC in SS, CX3CL1/CX3CR1 may become a new tool for the evaluation and diagnosis of SS.

In addition to these chemokines and their receptors, salivary gland ductal cells and inflammatory cells of SS patients showed reduced expression of CCL28 mRNA and increased expression of CCL21/CCR7, CCL5/CCR1 and CXCL1/CXCR2, accompanied by more severe histopathologic damage (72, 73). SS patients showed low levels of CCL2, but markedly high levels of CXCL17 and CXCL8 in their tears or saliva, with CCL2 associated with positive ocular tests (74–76). Another immunohistochemical study demonstrated that the ratio of CCL2 and CCL12 mRNA transcripts in the lacrimal gland lesion cells of MRL/MpJ mice increased with age and disease extent (77). Furthermore, the expression levels of CXCL9, CXCL10, CXCL11, and CXCR3 also increased in the tear film and ocular surface of SS patients, with the levels of CXCL11 and CXCL10 correlating with worsening ocular symptoms (78). CCL22 and CCL17 were detected in human salivary gland ductal epithelial cells by immunohistochemical analysis, and CCR4 was detected on lymphocytes heavily infiltrated in the glands (79). CCL19/CCR7 expression was found to be up-regulated in the salivary glands of SS patients compared to non-SS (61). Subsequently, in SS model mice, CCL22 was found to enhance the migratory activity of CD4+ T cells by increasing the expression of CCR4 on T cells to enhance salivary gland T cell responses (80). CXCL9 and CCL19 expression were significantly upregulated in the lacrimal glands of NOD mice, and their expression was regulated by IFN signaling (81). The chemokines and their receptors mentioned in the available literature and their main findings in SS patients (**Table 1**) and murine models (**Table 2**) are listed.

**Table 1:** Chemokines and the predominant cell types they recruit as pertinent in patients with SS.

Chemokines		Source cells	Receptors	adhesion molecules	Effector cells	Tissues and organs infiltrated	Related Cytokines	Related Pathways	Main findings	Reference	
CCL2	MCP-1	endothelial cells	CCR2	ND	T cells	lacrimal gland	ND	ND	↓CCL2 in SS tear and correlates with positive ocular tests.	(74)
CCL3/4	MIP-1α/β	macrophage derived DCs	CCR1, CCR5	ND	T,NK cells	salivary gland	ND	ND	↑CCL3/4 protein expression in association with GC-like structures.	(82)
CCL5	RANTES	macrophage derived DCs	CCR1	ND	T,NK cells	salivary gland	ND	ND	↑mRNA expression of CCL5/CCR1 in pSS circulating B cells.	(66)
CCL11	Eotaxin-1	endothelial cells	CCR3	ND	B cells,eosinophil	salivary gland	ND	ND	↑serum CCL11 expression is associated with GC-like structures.	(53)
CCL17	TARC	macrophages	CCR4	ND	Th2 cells	salivary gland	ND	ND	↑CCL17/CCR4 infiltration in SS salivary gland.	(79)
CCL19	ELC	DCs	CCR7	ND	B cells	salivary gland	BAFF	LTBR	↑CCL19/CCR7 expression in the salivary glands of SS patients compared to non-SS.	(61)
CCL21	SLC	DCs	CCR7	L-selectin/PNAD	T cells	salivary gland	ND	ND	↑CCL21/CCR7 expression is associated with anti-SSA/SSB titers, elevated ESR, IgG, RF and ESSDAI.	(61, 62)
CCL22	MDC	Macrophages, endothelial cells	CCR4	ND	Th2 cells	salivary gland	ND	ND	↑CCL22/CCR4 infiltration in SS salivary gland.	(80)
CCL25	TECK	epithelial cells	CCR9	α4β7/MAdCAM	Th17 cells	salivary gland,gut	IFN-γ, IL-17	PI3K/AKT	↑CCL25 and CCR9-expressing Th cells in the SS salivary gland.	(43, 65)
CCL28	MEC	epithelial cells	CCR10	ND	CD4+T cells	salivary gland	ND	ND	↓CCL28 in SS serum.	(72)
CXCL1	GRO-α	macrophages,epithelial cells	CXCR2	ND	Tregs,neutrophils	salivary gland	ND	ND	↑CXCL1/CXCR2 expression in the SS salivary gland.	(73)
CXCL8 (human only)	IL-8	macrophages, Endothelial cells	CXCR1	ND	CD8+T,DCs,neutrophils	salivary gland	ND	ND	↑CXCL8 in SS tears and saliva.	(75)
CXCL9	MIG	macrophages	CXCR3	ND	NK,CD8+T,Th1 cells	salivary gland	IFN-γ	JAK/STAT	↑CXCL9/10/11 and CXCR3 expression on ocular surface in SS.	(78)	
CXCL10	IP-10										
CXCL11	I-TAC										
CXCL12	SDF-1	DCs	CXCR4	LFA-1/ICAM-1	B cells	lacrimal gland	TGF-β	NF-κB	↑CXCL12 in MALT lymphoma and is associated with infiltrating epithelial and malignant B-cell components.	(56, 59)
CXCL13	BLC	follicular DCs	CXCR5	VLA-4/VCAM-1	Tfh,B cells	Salivary gland,lacrimal gland	TNF-α, IFN-γ	JAK/STAT, NF-κB	↑CXCL13/CXCR5 expression in association with lymphocytic infiltration and GC-like structures.	(50, 51)
CXCL17	DMC/VCC-1	DCs	CXCR8	ND	T cells	lacrimal gland	IL-18, IL-12	p38/MAPK	↑CXCL17 in SS tears and saliva.	(76)
CX3CL1	Fractalkine	macrophage, endothelial cells	CX3CR1	ND	NK,Th1 cells	salivary gland	IFN-γ	ND	↑CX3CL1/CX3CR1 expression in association with lymphocytic infiltration and GC-like structures.	(68–71)

ND, not determined.

**Table 2:** Chemokines associated with murine models of Sjögren’s-like disease and their chemotactic effects.

Chemokines	Murine models	Source cells	Receptors	Effector cells	Tissues and organs infiltrated	Related Cytokines	Related Pathways	Main findings	Reference
CCL2	MRL/MpJ mice	Macrophage,DCs	CCR2	Th2 cells	lacrimal gland	IL-4	ND	↑CCL2 mRNA transcript ratio in the lacrimal gland.	(77)
CCL3/4/5	NOD mice	DCs	CCR5	Th1 cells	salivary gland	IL-12	ND	↓CCR5 expression was accompanied by an increase in inflammatory chemokines.	(83)
CCL12 (mouse only) MCP-5	MRL/MpJ mice	monocytes	CCR2	Th2 cells	lacrimal gland	IL-4	ND	↑CCL12 mRNA transcript ratio in the lacrimal gland.	(77)
CCL17/22	MRL/MpJ mice	macrophages	CCR4	Th2 cells	lacrimal gland	IL-4	ND	Presence of CCL17 and CCL22 in the lacrimal gland.	(77)
CCL19/21	C57BL/6 mice	DCs	CCR7	B cells	salivary gland	BAFF	LTBR	↑CCL19 and CCL21 mRNA precedes the development of ELSs.	(84)
CXCL9/10	NOD mice	macrophages	CXCR3	Th1 cells	lacrimal gland	IFN-γ	JAK/STAT	↑CXCL9 and CXCL10 expression in the lacrimal gland.	(81)
CXCL12	aly/aly mice	macrophages,DCs	CXCR4	CD4+T cells	salivary gland	TGF-β	NF-κB	↑CXCL12 expression in salivary gland.	(60)
CXCL13	NOD mice;MRL/MpJ mice	macrophages, DCs	CXCR5	B cells	salivary gland	IFN-γ	JAK/STAT	↑CXCL13 expression in association with disease progression.	(85)
CX3CL1	NFS/sld mice	endothelial cells	CX3CR1	CD4+T cells	lacrimal gland	IFN-γ	ND	↑CX3CL1 in the lacrimal gland.	(86)

ND, not determined.

## 6 Lymphocyte homing and SS extra-glandular damage

SS is a diffuse connective tissue disease that can involve all systems of the body, and may present with extra-glandular organ involvement, such as interstitial lung disease (ILD), interstitial nephritis, annular erythema, primary biliary cirrhosis, and arthritis. This extra-glandular organ involvement may be related to lymphocyte leakage to the corresponding tissues and organs. Bronchus-associated lymphoid tissue is common in patients with SS lung complications, and the development of this tissue is associated with increased expression of lymphoid tissue chemokines such as CXCL13 and CCL21 (87). In mice injected with the stimulator of interferon gene agonist that showed lymphocytic infiltration in the peri-bronchial regions, the lungs showed increased expression of multiple chemokines (88). The atypical chemokine receptor CCX-CKR regulates the bioavailability of CCL19, CCL21, and CCL25. Lymphocyte infiltration has been observed in the salivary glands and liver of CCX-CKR gene-deficient mice with an increased incidence of SS-like liver lesions (89). SS patients with annular erythema showed strong expression of LFA-1/ICAM-1 on endothelial cells and monocytes, which may explain why most lymphocytes are localized around blood vessels (90). Additionally, LFA-1 and ICAM-1 showed higher expression in activated T cells in the joint fluid of patients with SS-associated arthritis (91). SS patients often present with reduced corneal innervation and corneal epithelial cell proliferation. Corneal epithelial cell proliferation in SS model mice has been reported to be associated with increased expression of CXCL1 in corneal epithelial mRNA (92). Another case-control study noted that female patients with SS showed elevated levels of CXCL10 in endocervical swab samples and increased lymphocytic infiltration in the vagina, which may account for vaginal dryness (93).

## 7 Lymphocyte homing targeted therapy in SS

Recent advancements in the field of lymphocyte homing have provided new insights into the pathogenesis of SS. Consequently, antibodies to cell adhesion molecules, antagonists of chemokines and their receptors, compounds that interfere with chemokine receptor signaling, and gene therapies targeting chemokines and their receptors have been gradually identified, providing new targets for human treatment of SS.

Retinoic acid is an important factor in maintaining intestinal homeostasis through direct regulation of effector cytokines (94). Inhibition or upregulation of retinoic acid leads to differential expression of CCR9 and α4β7 in Th17 cells (95). There are data suggesting that SS patients have lower retinoic acid concentrations and higher IL-17 expression levels in their serum compared to healthy controls, and NOD mice treated with retinoic acid showed reduced numbers of α4β7-negative Th17 cells and lower levels of IL-17 in vivo (43). These findings demonstrate the therapeutic potential of retinoic acid in SS, but more clinical validation is needed.

### 7.1 Antibodies to cell adhesion molecules

A study evaluating the efficacy of blocking cell adhesion molecules *in vivo* found that the combination of the ICAM-1 antibody with the anti-LFA-1 antibody prevented autoimmune lacrimal gland disease in SS model mice (96). Thus, the ICAM-1/LFA-1 pathway may play a key role in the development of T-cell-mediated inflammation in the lacrimal gland. On the basis of this finding, another study introduced a new tool, the multivalent biopolymeric nanoparticle (IBP-SI), which can inhibit the adhesion of lymphocytes to cells expressing high levels of ICAM-1 *in vitro*, disrupt the interaction between ICAM-1 and LFA-1, and can be utilized as an *in vivo* assessment tool or a potential therapeutic agent (46).

### 7.2 Anti-TNF-α treatment

TNF-α is a pro-inflammatory cytokine that is secreted in high amounts in many autoimmune or inflammatory diseases such as RA, Crohn’s disease, and SS (97). TNF-α promotes the expression of pro-inflammatory cytokines such as CXCR3, and promotes lymphocyte infiltration into the target organs, leading to the development of these diseases (98). TNF-α also destroys glands by inducing apoptosis of human salivary gland cells either alone or in combination with other inflammatory cytokines (49). Among patients with SS, TNF-α expression is elevated in salivary glands and serum (99). TNF-α-dependent activation of the exogenous apoptotic pathway was found to lead to upregulation of ICAM-1 and CCL20 in human salivary gland endothelial cells *in vitro*, which in turn promoted the infiltration of tissues by immune cells, mainly CD8+ lymphocytes (49). A study using SS model mice showed that TNF-α expression in the submandibular gland increased with the spontaneous development of SS-like exocrinopathy in NOD mice, and was accompanied by major infiltration of T and B cells (100). Thus, TNF-α may be an important pathogenic mediator in the pathogenesis of SS. Laboratory studies suggest that anti-TNF-α treatment may have a therapeutic effect on SS (100, 101). NOD mice administered neutralizing anti-TNF-α antibodies during the pre-morbid phase of the disease showed a marked improvement in salivary secretion, suggesting that the clinical signs of SS were alleviated (102). Another study in NOD mice revealed that TNF-α blockers could reduce the number of infiltrating T and B cells in the submandibular gland by downregulating the local expression of CXCL9 and CXCL13, and thereby alleviate glandular injury (100). Nevertheless, the findings from clinical trials investigating the efficacy of anti-TNF-α treatment in SS have been disheartening. Several randomized, double-blind, placebo-controlled studies examining the effectiveness of anti-TNF drugs such as infliximab and etanercept have failed to demonstrate any evidence of their efficacy in SS (103, 104). Consequently, further investigation is warranted to ascertain the potential usefulness of anti-TNF therapy in the clinical management of SS.

### 7.3 Inhibition of JAK/STAT signaling: baricitinib, CP-25

Type I interferon (IFN-α and IFN-β) and type II interferon (IFN-γ) target genes are upregulated in the peripheral blood and salivary glands of SS patients, and play central roles in SS pathogenesis (105). In the salivary glands of SS patients, DCs are the main source of IFN-α, whereas CD4+ T cells and NK cells are the main producers of IFN-γ (106). Recent studies have shown that IFN-α and IFN-γ are involved in the aggregation of infiltrating immune cells in the salivary glands of SS patients through the Janus kinase (JAK)-signal transducer and activator of transcription (STAT) signaling pathway by promoting the production of CXCL13 and CXCL10, respectively (107, 108). The JAK family of cytoplasmic protein tyrosine kinases includes JAK1, JAK2, JAK3, and tyrosine kinase 2. JAK binds to type 1 and type 2 cytokine receptors and transmits extracellular cytokine signals to activate STAT proteins, which translocate to the nucleus and regulate the transcription of effector genes (109). JAK-STAT signaling is initiated by over 50 cytokines binding to their respective receptors on the cell surface (110). Therefore, JAK is an excellent target for the treatment of various cytokine-mediated diseases. Baricitinib is a selective JAK1/2 inhibitor approved for the treatment of moderate-to-severely active RA (111). Baricitinib has been shown to inhibit IFN-γ-induced CXCL10 expression and attenuate immune cell chemotaxis through inhibition of JAK/STAT signaling (112). Very recently, the efficacy and safety of baricitinib for active SS patients have been explored in a pilot non-controlled trial. Baricitinib contributes to improving disease activity and main clinical manifestations, promising for the treatment of SS (113). The main active ingredient of total paeoniflorin is paeoniflorin (Pae), which has been used clinically for the treatment of autoimmune diseases (114). Paeoniflorin-6’-O-benzene sulfonate (CP-25) is a drug obtained by esterification of Pae that shows better lipid solubility and bioavailability than Pae (115). *In vitro* studies have revealed that CP-25 can negatively regulate the JAK1-STAT1/2 pathway, counteracting CXCL13 secretion and downregulating CXCL13 expression, and impede the migration of lymphocytes to the salivary glands (116, 117). These findings provide an experimental basis for the evaluation of CP-25 as a potential drug for the treatment of SS.

### 7.4 LTBR antagonists: baminercept, Rituximab

B cell-activating factor (BAFF), also known as B lymphocyte-stimulating factor, is involved in B cell survival and humoral immune response, and plays a key role in B cell homeostasis (118). Elevated levels of BAFF have been observed in the serum, saliva and salivary glands of SS patients (119). In addition, higher levels of BAFF have been observed in the salivary glands of patients with GCs (120). *In vivo* experiments confirmed that NOD mice treated with soluble BAFF receptor (BAFFR) and anti-CXCL13 antibody do not develop salivary dysfunction, and that blocking BAFFR attenuates SS disease progression and may be an effective therapeutic strategy for SS (121). The lymphotoxin-beta receptor (LTBR) pathway has been associated with the appearance of ELSs at sites of chronic inflammation in a variety of autoimmune diseases, and has been shown to regulate the expression levels of CXCL13, CCL19, and PNAd (122, 123). Evidence from animal models and SS patients suggests that CXCL13 and the LTBR receptor pathway are required for the development of ELSs and may be effective therapeutic targets for SS (124). Blockade of LTBR reduced CXCL13 protein expression in the lacrimal gland of NOD mice, and CCL19 expression was significantly inhibited in the infiltration zone of salivary glands (125). LTBR antagonists inhibited HEV addressin expression and lymphocyte infiltration in the diseased glands, with beneficial effects on tear and saliva secretion and the integrity of the ocular surface (126). Baminercept is a lymphotoxin-beta receptor IgG fusion protein (LTBR-Ig). A multicenter trial suggested that baminercept significantly reduced the plasma levels of CXCL13 and altered the number of circulating B and T cells in SS patients, but failed to significantly improve glandular and extra-glandular disease in SS patients (127). Further observations with expanded samples are needed in future studies. Rituximab (RTX), a chimeric humanized monoclonal anti-CD20 antibody, inhibits B cell activation, proliferation and differentiation, and acts as a B cell scavenger. A prospective, multicenter, follow-up study demonstrated that B cell depletion therapy with RTX could restore B cell disorders by decreasing salivary gland LTB mRNA levels, modulating CXCL13/CXCR5 interactions, eliminating ELSs, and decreasing immune infiltration and lymphoid organization in target tissues (128). However, a double blind study has also demonstrated that RTX does not yield a substantial improvement in SS-related symptoms and immunological parameters of SS, and that the association is primarily limited to fatigue (129). Hence, further researches are justified to clarify the therapeutic efficacy of RTX in the treatment of SS.

### 7.5 Blockade of NF-κB pathway: AMD3100, A20

NF-κB plays a key role in the regulation of many inflammatory processes of immune cells (130). Two NF-κB signaling pathways exist in the immune cells: a classical pathway initiated by the NF-κB1 complex and an alternative nonclassical pathway initiated by the NF-κB2 complex (131). A previous study showed that activation of the NF-κB2 pathway in (aly)/aly mice negatively regulates TGF-β signaling, whereas TGF-β upregulates the expression of CXCR4 and regulates the migration of T cells toward autoimmune targets, leading to damage similar to that seen in the early histopathological stages of SS (132). The CXCR4 antagonist AMD3100 inhibits autoimmune lesions in (aly)/aly mice by reducing TEM cell infiltration (60). Knockdown of the NF-κB pathway inhibitor A20 in mouse submandibular gland epithelial cells resulted in upregulation of CXCR4 expression, triggering T lymphocyte infiltration and formation of immune foci (133). NF-κB2 controls the migratory activity of TEM by regulating the expression of CXCR4, which may be a potential therapeutic target for the treatment of SS.

**Figure 1:**
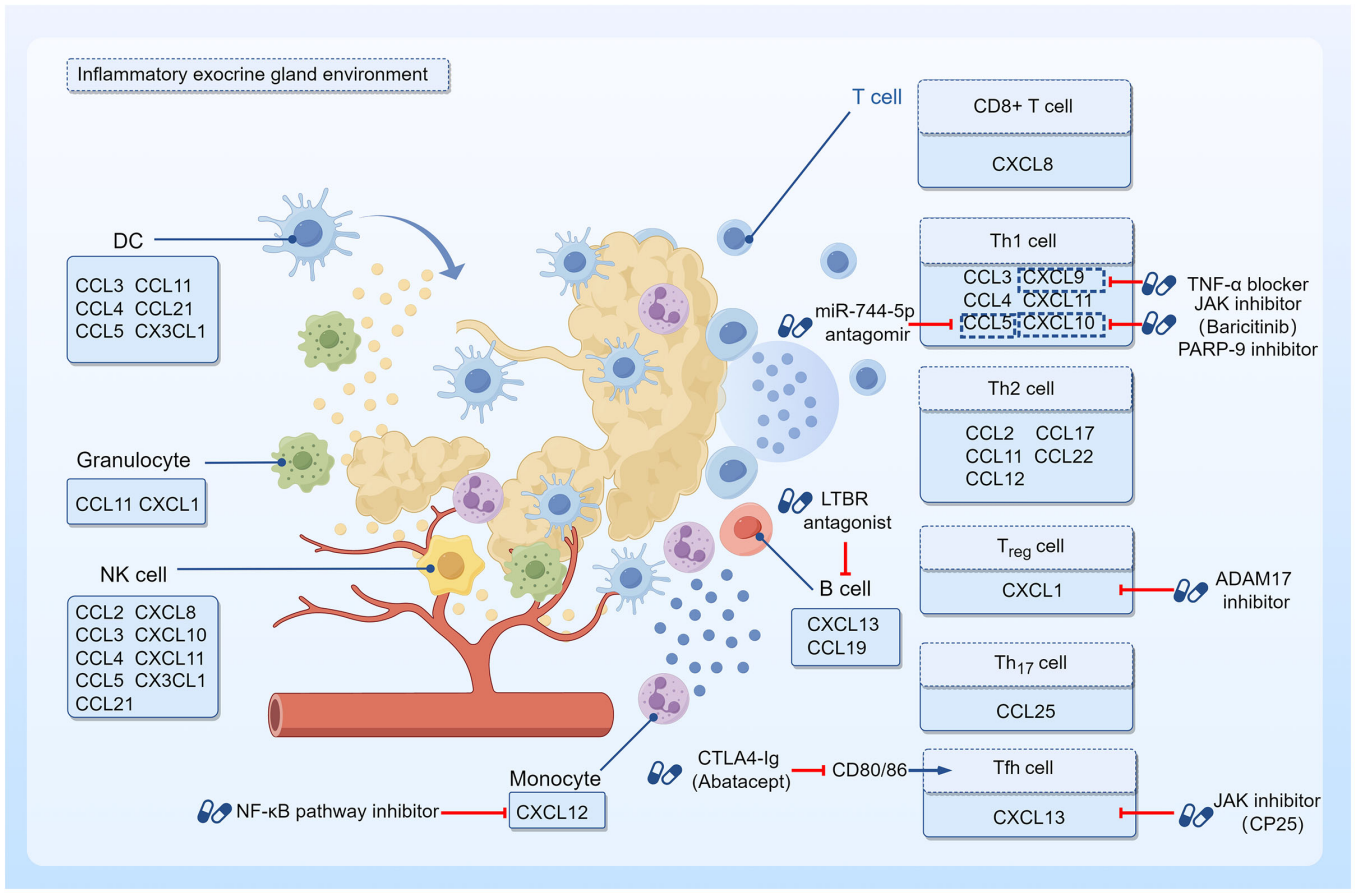
Targeted therapy with chemokines and their effector cells. The inflammatory environment of SS exocrine glands shows infiltration of T and B lymphocytes around ductal cells with a few other immune cells such as monocytes, granulocytes, DCs, and NK cells. As chemokine effector cells, they are able to express specific chemokine receptors that bind to the corresponding chemokines, thus showing chemotactic properties. Drugs with the potential to prevent chemokine effector cells from infiltrating into tissues and organs have been investigated on this basis. Jak inhibitor CP-25 could downregulate CXCL13 expression, and impede the migration of lymphocytes to the salivary gland. Baricitinib, another Jak inhibitor, suppressed IFN-γ-induced CXCL10 expression and attenuated immune cell chemotaxis. PARP-9 inhibitor also downregulated CXCL10 and reduced immune cell infiltration during SS progression. CTLA4-Ig treatment inhibited CD80/86 and reduced the number of activated circulating Tfh cells. LTBR antagonists reduced CXCL13 protein expression. NF-κB pathway inhibitors downregulated CXCL12 receptor expression in monocytes. TNF-α blockers could downregulate the local expression of CXCL9. The use of a miR-744-5p antagomir decreased the levels of the CCL5 in the inflammatory milieu. ADAM17 inhibitors abrogated CXCL1/CXCR2 interactions to block the inflammatory response.

Notably, progress in the field of lymphocyte homing has led to the development of novel targets and drugs with therapeutic potential. For example, abatacept treatment reduces the number of activated circulating Tfh cells, thereby contributing to the attenuation of Tfh cell-dependent B cell hyperactivity in SS (134); disintegrin and metalloproteinase-17 (ADAM17) inhibitors abrogate CXCL1/CXCR2 interactions to block the inflammatory response in SS (135); miR-744-5p may be a potential therapeutic target to ameliorate ocular inflammation in SS patients with dry eye (136); and poly (ADP-ribose) polymerase family member 9 (PARP-9) is a regulator of immune cell infiltration during SS progression (137). Nevertheless, most of these candidates are still in the preclinical stage, and more research efforts are needed to obtain evidence for their use in SS treatment. In summary, the development of new targets that can modulate lymphocyte migration molecules is a promising strategy for the treatment of SS (**Figure 1**).

## 8 Conclusions

Lymphocyte homing is a complex process involving multiple molecules, and the migration associated with this process is extensive and multisite. SS pathogenesis is mainly related to the abnormal infiltration of lymphocytes into exocrine glands, and SS treatments targeting lymphocyte homing are a hotspot of current research. In the pathogenesis of SS, the interaction of lymphocyte homing receptors and addressins such as L-selectin/PNAD, α4β7/MAdCAM-1, VLA-4/VCAM-1, LFA-1/ICAM-1, and chemokines and their receptors such as CXCL13/CXCR5, CXCL12/CXCR4, CCL21/CCR7, CCL25/CCR9, CX3CL1/CX3CR1 regulate lymphocyte migration to the corresponding tissues and organs. Studies have shown that a variety of molecules are involved in lymphocyte homing, including TNF-α, IFN-α, IFN-β, and BAFF, and the process mainly involves the JAK-STAT signaling pathway, the LTBR pathway, and the NF-κB signaling pathway. Blocking lymphocyte homing can alleviate the disease, but considering the complexity of this process, the specific molecular mechanisms need to be explored more deeply to identify more specific molecules to provide targeted therapy for SS treatment.

## Author contributions

JHL: Data curation, Writing – original draft. X-BY: Writing – original draft. ZH: Writing – original draft. QH: Writing – original draft. JY: Writing – original draft. YZ: Writing – original draft. JC: Writing – review & editing. WS: Writing – review & editing. JL: Conceptualization, Writing – review & editing. QT: Conceptualization, Writing – review & editing.
